# The Health-Benefits and Phytochemical Profile of *Salvia apiana* and *Salvia farinacea* var. *Victoria Blue* Decoctions

**DOI:** 10.3390/antiox8080241

**Published:** 2019-07-25

**Authors:** Andrea F. Afonso, Olívia R. Pereira, Ângela S. F. Fernandes, Ricardo C. Calhelha, Artur M. S. Silva, Isabel C.F.R. Ferreira, Susana M. Cardoso

**Affiliations:** 1 QOPNA & LAQV-REQUIMTE, Department of Chemistry, University of Aveiro, 3810-193 Aveiro, Portugal; 2 Public Health Laboratory of Bragança, Local Health Unit, Rua Eng. Adelino Amaro da Costa, 5300-146 Bragança, Portugal; 3 Centro de Investigação de Montanha (CIMO), Instituto Politécnico de Bragança, Campus de Santa Apolónia, 5300-253 Bragança, Portugal

**Keywords:** sage, bioactivity, antioxidant, anti-inflammatory, cytotoxicity, antibacterial, phenolic compounds, terpenes

## Abstract

*Salvia apiana* and *Salvia farinacea* var. *Victoria Blue* decoctions were screened for diverse bioactivities, including the ability to counteract oxidative and inflammatory events, as well as to act as cytotoxic and antimicrobial agents. Both extracts showed good activities and that of *S. apiana* origin was particularly effective regarding the ability to prevent lipid peroxidation and to prevent nitric oxide (NO●) production in lipopolysaccharide (LPS)-activated murine macrophage RAW 264.7 cell line (EC_50_ = 50 μg/mL). Moreover, it displayed high cytotoxic capacity against hepatocellular carcinoma HepG2, cervical carcinoma HeLa, and breast carcinoma cells MCF-7, but comparatively low effects in porcine liver primary cells, which highlights its selectivity (GI_50_ = 41–60 μg/mL vs. 362 μg/mL, respectively). Further, it exhibited inhibitory and lethal potential against a panel of Gram-positive and Gram-negative bacteria. It is possible that the bioactive properties of the two *Salvia* extracts are associated to their phenolic components and, in the particular case of *S. apiana*, to its richness in phenolic terpenes, namely in rosmanol, hydroxycarnosic acid and a derivative of sageone, which were found in the extract.

## 1. Introduction

In normal physiological conditions, cells maintain a redox homeostasis, i.e., the balance between reactive species formation and their elimination. Still, when equilibrium is disrupted, oxidative stress is settled and the overproduction of reactive species causes several mitochondrial and cellular damages, namely in lipids, proteins, DNA, and other macromolecules. Ultimately, these events are known to be associated with aging and the onset and/or development of distinct diseases, including cancer [1,2]. In this context, diet supplementation with plant-based products rich in phytochemicals are believed to counteract oxidative-related events, thus contributing to human health [1,3,4].

*Salvia* is the largest genus of the Lamiaceae family, with over 900 species [5] claimed for their richness in essential oils and/or phenolic compounds [6,7,8,9], a fact that is closely associated to their usage in traditional medicine and potential applications in distinct industries, including food, pharmaceutical, and cosmetics [4,10]. *Salvia farinacea* var. *Victoria Blue* and *Salvia apiana* are two edible *Salvia* plants native to southwestern regions of the USA and Mexico. The first is a perennial shrub that typically grows to be 45–90 cm, whose gray-green leaves are drooping, irregularly-serrate, ovate-lanceolate, and contain a compact multi-branched rich violet-blue flowers spikes, resembling lavender [11,12,13]. In turn, *S. apiana*, also known as white sage, is a branched shrub growing up to 1.5 m, with silvery white petiolate leaves highly aromatic, and white to pale lavender flowers [14,15].

Although lowly exploited, *S. apiana* has been previously studied regarding its essential oils composition, which are known to contain high amounts of terpenes and terpene derivatives, particularly β-pinene, α-pinene, borneol, 1,8-cineole, campholenic acid, and β-caryophyllene [13,16]. Moreover, phytochemical analysis of hydroethanolic or acetone extracts of this species have allowed the identification of distinct diterpenes and triterpenes, as well as the flavonoids cirsimaritin and salvigenin [17,18]. Still, as far as we know, the biological properties of polar extracts from *S. apiana* aerial parts were limited to the screen of the cannabinoid or opioid receptors activity of an ethanolic extract [17] and the potential cytotoxicity against leukemia cell lines of a methanolic extract [19]. With regard to *S. farinacea* var. *Victoria Blue*, both chemical and biological properties of polar extracts remain unexplored. Therefore, the present study aimed to investigate potential biological effects of *S. apiana* and *S. farinacea* var. *Victoria Blue*, having in mind the traditional usage of *Salvia* species, which have been used for centuries in the form of infusions and decoctions. The antioxidant, anti-inflammatory, cytotoxicity, and antibacterial potencies of decoctions will be associated with their specific phenolic components.

## 2. Materials and Methods

### 2.1. Chemicals

Rosmarinic acid and the 7-*O*-glucoside derivatives of apigenin, luteolin, eriodyctiol, quinic acid, caffeic acid, 5-*O*-caffeoylquinic acid, 4-*O*-hydroxybenzoic acid, salvianolic acid B, caffeic acid, ferulic acid, and 4-*O*-coumaric acid were obtained from Extrasynthese (Genay Cedex, France). Trolox, sulforhodamine B (SRB), DPPH (2,2-diphenyl-1-picrylhydrazyl) radical, acetic acid, ellipticine, trypan blue, trichloroacetic acid (TCA), Tris, lipopolysaccharide (LPS), carnosol, nisin, ascorbic acid, and butylated hydroxyanisole (BHA) were obtained from Sigma Chemical Co (St Louis, MO, USA). Fetal bovine serum (FBS), L-glutamine, trypsin-ethylenediaminetetraacetic acid (EDTA), penicillin/streptomycin solution (100 U/mL and 100 mg/mL, respectively), Roswell Park Memorial Institute (RPMI) 1640 Medium and Dulbecco’s Modified Eagle Medium (DMEM) were from Hyclone (Logan, Utah, UT, USA). The Griess reagent system was purchased from Promega Corporation (Madison, WI, USA). Mueller-Hinton agar was from VWR (Prolabo Chemicals, USA); formic acid and ethanol was from Panreac (Barcelona, Spain). *n*-Hexane, methanol, and acetonitrile were purchased from Lab-Scan (Lisbon, Portugal). Purified water was obtained from a Direct-Q^®^ water purification system (Merck Life Science, Darmstadt, Germany).

### 2.2. Plant Material

The *S. apiana* and *S. farinacea* var. *Victoria Blue* species were collected from the fields of Coimbra College of Agriculture, Portugal, GPS coordinates 40.211439, −8.451251. After collection, its aerial parts were dried in a ventilated incubator at 35 °C for 3 days and kept in a dark room until use.

### 2.3. Preparation of Extracts

Extraction of the phenolic compounds was performed by decoction, following a method previously described [20] with slight changes. Briefly, the aerial parts (flowers, leaves, and stems) of sage plants were boiled for 15 min (5 g of plant in 100 mL of water), filtrated, and concentrated to about half of the volume in a rotary evaporator, to reduce the amount of water, followed by defatting with *n*-hexane (1:1 *v/v*). Traces of *n*-hexane in the aqueous phase were eliminated by rotary evaporation before freeze-drying. The resulting extract was kept under vacuum in a desiccator in the dark and a stock water solution (10 mg/mL) was prepared just before the analysis.

### 2.4. Identification and Quantification of Phenolic Compounds

Ultra-high Performance Liquid Chromatography coupled to Diode Array Detector and an Electrospray Mass Spectrometer (UHPLC-DAD-ESI/MS^n^) analyses of phenolic profiles from the two decoctions (5 mg/mL) were carried out on an Ultimate 3000 (Dionex Co., San Jose, CA, USA) apparatus equipped with an ultimate 3000 Diode Array Detector (Dionex Co., San Jose, CA, USA) and a Thermo LTQ XL mass spectrometer (Thermo Scientific, San Jose, CA, USA), following a method previous described [21]. Gradient elution was carried out with a mixture of 0.1% (*v/v*) of formic acid in water (solvent A) and acetonitrile (solvent B). The solvent gradient used consisted of a series of linear gradients starting from 5% of solvent B and increasing to 23% at 14.8 min, to 35% at 18 min, and to 100% at 21 min over three minutes, followed by a return to the initial conditions. The mass spectrometer used was a Thermo Xcalibur Qual Browser (Thermo Scientific, San Jose, CA, USA) and the operations were carried out using the conditions previously described [22]. Quantification was performed by the external standard method using the calibration curves of structurally-related standard compounds. Considering the nature of the phenolic compounds, their quantification was performed at 280, 320, or 340 nm, also considering the limit of detection (LOD) and limit of quantification (LOQ). LOD and LOQ were determined from the parameters of the calibration curves, being defined as 3.3 and 10 times the value of the regression error divided by the slope, respectively [21].

### 2.5. Antioxidant Activity

#### 2.5.1. DPPH● Scavenging Test

The extracts capacity for scavenging DPPH• was evaluated following the procedure previously described by Catarino et al. [23]. Ascorbic acid was used as positive control and the results were expressed as EC_50_ values (sample concentration providing 50% of antioxidant activity).

#### 2.5.2. Ferric Reducing Power Assay

The ability of the two sage extracts to reduce iron (III) to iron (II) was carried out according to a procedure described before [23], on which the antioxidant compounds from the extracts form a colored complex with potassium ferricyanide, trichloroacetic acid, and ferric chloride, measurable at 700 nm. BHA (2,6-di-*tert*-butyl-4-methylphenol) was used as positive control and the results were expressed as EC_50_ values.

#### 2.5.3. Thiobarbituric Acid Reactive Substances (TBARS)

The decrease in thiobarbituric acid reactive substances (TBARS) was evaluated by the inhibition of lipid peroxidation in porcine (*Sus scrofa*) brain homogenates, according to a procedure described by Martins et al. [24]. The color intensity of the malondialdehyde-thiobarbituric acid (MDA-TBA) was measured as its absorbance at 532 nm; the inhibition ratio (%) was calculated using the following equation: [(A−B)/A] × 100%, where A and B were the absorbance of the control and the sample solutions, respectively. Trolox was used as positive control and the results were expressed as EC_50_ values.

#### 2.5.4. β-Carotene Bleaching Assay

β-carotene linoleate general assay was performed as described by Barros et al. [25]. Trolox was used as positive control and the results were expressed as EC_50_ values.

### 2.6. Anti-Inflammatory Activity

The extracts ability in scavenging the NO radical was evaluated in the mouse macrophage-like cell line RAW 264.7 following the general procedure previously described [26]. Cells were treated under 5% CO_2_ in humidified air, using DMEM culture medium enriched with 10% heat inactivated fetal bovine serum, glutamine, and antibiotics at 37 °C. For the tests, cells were seeded in 96-well plates (150,000 cells/well) and allowed do attach to the plate overnight. Then, these were treated with extract solutions (concentration of 25 and 100 µg/mL, for each extract) for 1 h, followed by the stimulation with LPS (1 μg/mL) for 18 h. The effect of all the tested samples in the absence of LPS was also evaluated, in order to observe if they induced changes in nitric oxide (NO●) basal levels. Dexamethasone (50 µM) was used as a positive control while negative controls had no added LPS. The NO levels produced were determined by the Griess reaction, used for measuring the nitrite accumulation in the culture supernatant on macrophage cell line RAW 264.7 [26]. The anti-inflammatory activity of each extract was determined by calculating EC_50_ values (μg/mL), which corresponds to the sample concentration that provides 50% inhibition of NO production.

### 2.7. Cytotoxic Effect in Four Human Tumor Cell Lines

The cytotoxic effect of *S. apiana* and *S. farinacea* var. *Victoria Blue* extracts towards four human tumor cell lines were carried out by the sulforhodamine B (SRB) assay, using the conditions established by Souza et al. [26]. The tumor cell lines MCF-7 (breast adenocarcinoma), NCI-H460 (non-small cell lung cancer), HeLa (cervical carcinoma), and HepG2 (hepatocellular carcinoma), were maintained in enriched medium, at 37 °C, in a humidified air incubator containing 5% CO_2_. Each cell line was plated at an appropriate density (7.5 × 10^3^ cells/well for MCF-7 and NCI-H460 or 1.0 × 10^4^ cells/well for HeLa and HepG2) in 96-well plates. The cytotoxicity results were expressed as GI_50_ values (μg/mL), corresponding to sample concentration that inhibited 50% of the cell growth. Ellipticine was used as positive control.

### 2.8. Cytotoxic Effect in Non-Tumor Liver Cells

The cytotoxicity of the sage extracts were tested by hepatotoxicity assay, in a primary non-tumor cell culture obtained from porcine liver (PLP2) following the described procedures [26]. For the tests, cells were seeded (at 1.0 × 10^4^ cells/well) in enriched medium. Results were expressed as GI_50_ values (μg/mL) and ellipticine was used as a positive control.

### 2.9. Antimicrobial Activity

The antibacterial potential of the *S. apiana* and *S. farinacea* var. *Victoria Blue* species was evaluated against five bacterial strains, including Gram-positive bacteria (*Staphylococcus epidermidis* NCTC 11047 and *Staphylococcus aureus* NCTC 6571) and Gram-negative bacteria (*Salmonella typhimurium* NCTC 12023, *Escherichia coli* NCTC 9001, and *Pseudomonas aeruginosa* NCTC 10662) from the National Collection of Type Cultures, operated by Public Health England, Salisbury, United Kingdom. All strains were cultured in Mueller-Hinton agar and incubated at 37 °C for 24 h. The minimum inhibitory concentration (MIC) and minimum bactericidal concentration (MBC) of aqueous solutions of both *Salvia* species were determined by the broth microdilution method using an adapted method previous described by Afonso et al. [21]. MIC is defined as the lowest concentration at which visible growth is inhibit, while MBC is the lowest concentration of the tested substance which has a bactericidal effect. Briefly, bacterial suspensions were prepared by direct colony suspensions and a final inoculum of 1.5 × 10^5^ CFU/mL was required for final suspensions that was diluted in a 1:100 ratio in Mueller-Hinton broth. Next, 100 microliters of this medium was dispensed into wells of 96-well micro titer plates and *S. apiana* and *S. farinacea* var. *Victoria Blue* decoctions were added and serially diluted four times across the plate. Then, 100 microliters of bacteria suspension was added to each well and the plates were incubated at 37 °C for 24 h. The assay for each pathogen was repeated three times [27] MBC values are determined by sub-culturing from each negative well onto Mueller-Hinton agar and confirmation the lowest concentration with no visible growth [28]. Nisin was used as the positive control.

### 2.10. Statistical Analysis

The results were analyzed using GraphPad Prism 6 (GraphPad Software, CA, USA). Data were expressed as mean ± S.D. of 3–4 independent experiments performed at least in triplicate. One-way analysis of variance (ANOVA) followed by Tukey’s test was used to detect any significant differences among different means. The *p*-value less than 0.05 were assumed as significant difference.

## 3. Results and Discussion

### 3.1. Antioxidant Activity

Many plants, including *Salvia* species, have been evaluated for their claimed antioxidant capacities and/or ability to ameliorate oxidative-stress related disorders, as these properties are commonly associated to the richness in phenolic compounds [4,29].

The antioxidant activity of *Salvia* decoctions was evaluated by four generalized methods: DPPH●, ferric reducing power assay, bleaching of β-carotene, and TBARS. Globally, *S. apiana* decoction presented superior antioxidant capacity than that of *S. farinacea* var. *Victoria Blue*, a trend that was particularly evident with respect to lipid peroxidation events (Table 1), with EC_50_ values obtained of about one-eighth or the same magnitude of the commercial standard, in the TBARS and β-carotene bleaching assays, respectively. Regardless of being less active, it is important to note that *S. farinacea* var. *Victoria Blue* extract may also be considered as a promising antioxidant agent, since EC_50_ values in all tests were only 1.8–3.7-fold those of the standard compounds.

As far as we know, the antioxidant capacities of *S. apiana* and *S. farinacea* var. *Victoria Blue* polar extracts have not been exploited before. However, the promising abilities herein reported for decoctions agree and/or, in particular in the case of *S. apiana*, have exceeded those previously described for sage plants [6,7,30,31].

### 3.2. Anti-Inflammatory Aactivity

Nitric oxide produced in high concentrations by macrophages has been reported to be involved in inflammation and compounds targeting its production are considered as good candidates for attenuating inflammatory-related diseases [32]. In our study, the potency of *S. apiana* and *S. farinacea* var. *Victoria Blue* extracts to counteract NO● production was evaluated in an LPS-activated RAW 264.7 macrophage model. As shown in Table 2, both extracts were effective. Particularly, *S. apiana* was promising, as its potency corresponded to about one-third of the drug dexamethasone (EC_50_ = 49.9 ± 2.5 and 16.0 ± 1.0, respectively). Moreover, their activity seems to be superior to that described by Ravipati et al. [33] and Jang et al. [34] for commercial aqueous extracts of *Salvia miltiorrhiza* (EC_50_ = 200 μg/mL) and for the 80% ethanolic extracts of aerial parts and of roots of *Salvia plebeia* (EC_50_ between 500–1000 and superior to 1000 μg/mL), albeit that the absence and/or use of a different reference compound by the authors hamper more solid conclusions. In addition, no relationship was made by the authors between the anti-inflammatory effects and the possible compounds involved.

### 3.3. Cytotoxic Activity

The effect of the two *Salvia* decoctions on the growth of tumor and non-tumor cell lines was evaluated by the SRB assay. In general, both *Salvia* extracts showed promising cytotoxic effects against tumoral cell lines, particularly hepatocellular carcinoma HepG2, cervical carcinoma HeLa, and breast carcinoma cells MCF-7 (Table 2). Further, between the two extracts, *S. apiana* exhibited more cytotoxicity than *S. farinacea* var. *Victoria Blue*, a fact that was clear in HepG2 cells (GI_50_ of 40.9 ± 3.3 vs. 87.4 ± 5.4 μg/mL, respectively) and HeLa (GI_50_ of 57.3 ± 5.1 vs. 77.8 ± 3.5 μg/mL, respectively). Note that the data seems to agree with the results of Saeed et al. [19], who reported a high cytotoxic effect of *S. apiana* methanolic extracts against both sensitive and multidrug-resistant leukemia cells (IC_50_ values of 7.17 ± 0.67 and 9.91 ± 0.8 μg/mL, respectively), as assessed by the resazurin reduction assay. Although our results cannot be directly compared to those reported previously for other plants (due to the absence or use of different standards or methodologies), they generally reinforce the theory that polar extracts of sage plants may act as cytotoxic agents. In fact, other authors have previously highlighted cytotoxic effects for such extracts, e.g., *Salvia eremophila* methanolic or hydromethanolic extracts, which were effective in breast cancer MCF-7 cell lines (IC_50_ values of 47.7 and 75.2 μg/mL, respectively), with 7 to 11 fold less potency of the drug cisplatin [35]. Using the MTS assay, Jiang et al. [36] also highlighted a high cytotoxicity capacity of ethanolic and acetone extracts from *S. miltiorrhiza* roots against HepG2 cell lines (IC_50_ = 17.3 and 83.2 µg/mL, respectively), as well as of *Salvia officinalis* roots and leaves (IC_50_ = 19.6–43.8 µg/mL and 64.4–90.0 µg/mL, respectively).

Moreover, our results also indicated that the anti-proliferative activity of *S. apiana* and *S. farinacea* var. *Victoria Blue* decoctions were tumor-selective, as reflected by comparatively low GI_50_ values in the cancer cell lines with regard to those of PLP2 cells (GI_50_ = 361.7 and 335.4 μg/mL, respectively). This ability to discriminate tumor cells is essential to minimize cytotoxic effects in normal cells, and has also been described for other *Salvia* polar extracts, including *S. officinalis*, which regardless being cytotoxic to MCF-7 cancer cells (IC_50_ = 142.4 μg/mL), showed no significant effects against normal human umbilical vein endothelial cell cells (IC_50_ values above 600 µg/mL) [37].

### 3.4. Antibacterial Activity

The antibacterial potential of the two *Salvia* decoctions towards the Gram-positive bacteria *S. aureus* and *S. epidermidis* and the Gram-negative bacteria *S. typhimurium*, *E. coli* and *P. aeruginosa* are summarized in Table 3. In general, *S. apiana* had promising antibacterial properties, with inhibitory and lethal capacity at concentrations equal or below to 0.69 mg/mL (for *S. aureus* and *S. epidermidis*) and of 2.75 mg/mL (for the *S. typhimurium*, *E. coli*, and *P. aeruginosa)*, which corresponded to only 3–5 times less that of the drug nisin. This contrasted with *S. farinacea* var. *Victoria Blue* decoction, that only showed relevant antibacterial effect towards the Gram-positive bacteria *S. aureus* (MIC and MBC of 1.06 and 2.12 mg/mL, respectively).

As far as we know, the antibacterial effects of *S. apiana* and *S. farinacea* var. *Victoria Blue* polar extracts have not been previously exploited. Still Córdova-Guerrero et al. [38] has remarked the antimicrobial activity of a *S. apiana* hexane extract against Gram-positive bacteria, using the agar disc diffusion assay, for which they obtained an inhibition hale of *S. aureus* in the range of 10 to 24 mm at 0.34–2.7 µg/µL. Unfortunately, as the authors did not present the MIC value, a direct comparison to our results is not possible. Nonetheless, other authors have previously described promising antimicrobial effects of polar extracts of sage species. For example, Ibrahim et al. [39] described a powerful activity of *Salvia bicolor* methanolic extract against a distinct Gram-positive bacteria, especially on *S. aureus* and *S. epidermidis* (MIC values of 0.2 and 0.35 mg/mL, respectively) and Gram-negative bacteria (MIC values in the range 0.4 to 1 mg/mL) [39], a fact that the authors associated with its richness in phenolic compounds, which were mostly gallic, *p*-coumaric and protocatechuic acids, and flavonoids (e.g., luteolin-7-*O*-glucoside). Moreover, when screening the antimicrobial potential of hydromethanolic extracts of different *Salvia* species through the nutrient-broth microdilution assay, Firuzi et al. [35] reinforced that *S. eremophila, Salvia limbata, Salvia santolinifolia,* and *Salvia sclarea* extracts were able to inhibit the growth of a panel of six microorganisms, with MIC values in the range of 0.31–5 mg/mL.

### 3.5. Characterization of the Extracts

**Figure 1:**
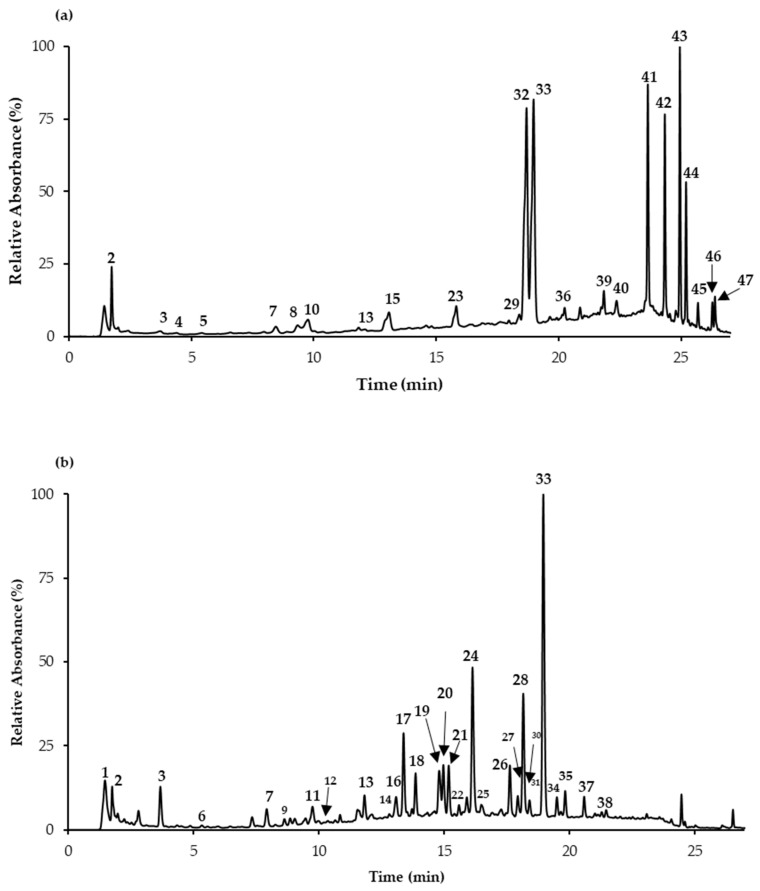
Chromatographic representation of *Salvia* decoctions at 280 nm: (**a**) *S. apiana*; (**b**) *S. farinacea* var. *Victoria Blue*. Numbers in the figure correspond to the UHPLC-DAD-ESI-MS^n^ peaks described in Table 4.

Not surprisingly, the levels of phenolic compounds in *Salvia* decoctions were significantly different, with that from *S. apiana* being about six times richer than *S. farinacea* var. *victoria* (Table 4). Moreover, regardless of being rich in rosmarinic acid (56.8 and 17.8 mg/g extract, respectively) as in general for polar extracts from *Salvia* plants, the two extracts differed with regard to their phenolic constituents, as well as to those previously reported for *Salvia elegans*, *Salvia greggii,* and *S. officinalis* decoctions [40]. Indeed, *S. apiana* decoction was characterized by its richness in terpenes derivatives (peaks 41 and 43–47, in Figure 1 and Table 4), which were mainly represented by rosmanol ([M−H]^−^ at *m/z* 345 → 301, 271, 283), a derivative of sageone ([M−H]^−^ at *m/z* 361) and hydroxycarnosic acid ([M−H]^−^ at *m/z* 347 → 303, 273), with estimated levels of 192.4 ± 17.1, 174.1 ± 14.1 and 69.7 ± 11.2 mg/g extract, respectively, and other less abundant ones, including carnosol, carnosic acid, and tetrahydrohydroxyrosmariquinone (peaks 45, 46, and 47, respectively), in addition to some flavonoids (hesperidin, quercetin-*O*-hexoside, and cirsimaritin). These results strengthen those previously reported [17,18,41], allowing to conclude that polar extracts from *S. apiana* are particularly rich in phenolic terpenes. It should be noted that among the compounds herein detected in the decoction of *S. apiana*, rosmanol, and hydroxycarnosic acid were previously described in the acetone extracts [18], while carnosol, sageone, and carnosic acid were reported in aqueous ethanolic and/or methanolic extracts from the same plant [17,41]. 

Although less rich in phenolic compounds, in addition to rosmarinic acid, *S. farinacea* var. *Victoria Blue* decoction presented moderate quantities of common flavones to the *Salvia* plants, namely apigenin-*O*-hexoside (peak 28, 16.7 mg/g extract) and luteolin-*O*-glucuronide (peak 24, 15.8 mg/g extract) [42,43,44].

Thus, our study suggests that the high bioactivity of *S. apiana* decoction is associated with its fertility in phenolic components, namely flavonoids and terpene derivatives. In fact, the diterpene rosmanol (the most predominant compound in *S. apiana* extract) and its derivatives were previously associated with the high antioxidant potential of *S. officinalis* [45,46] and the cytotoxic activity of several other *Salvia* plants [8]. In addition, sageone is an abietane diterpenoid reported to occur in *S. apiana* species [17] and described to exert antiviral activity [8]. Moreover, others, including carnosol and carnosic acid, are claimed to exert several biological properties, including lipase inhibitory activity [47], antioxidant, antimicrobial, anti-inflammatory, and antitumoral [45,46,48]. Naturally, the possible contribution of other components of the extract, including cirsimaritin, which have already been considered relevant in the antibacterial and antioxidant activities of *Salvia* species [8,46] should not be overlooked.

## 4. Conclusions

The antioxidant, anti-inflammatory, cytotoxic and antibacterial properties of *S. apiana* and *S. farinacea* var. *Victoria Blue* decoctions were reported in the present work, allowing us to conclude that both have potential bioactive effects, which was extremely promising, particularly with regard to the counteract of key events in oxidative stress, showing EC_50_ values of 2.79 µg/mL and 41.2 µg/mL in TBARS and β-carotene bleaching assays, respectively. Decoction of *S. apiana* also had an effect on the suppression of inflammatory events, since it displayed a third of the potency of the drug dexamethasone to inhibit NO● production by LPS-activated RAW 264.7 macrophages. In addition, this decoction was selective for tumors, exhibiting cytotoxic capacity in cancer cell lines with low cytotoxic effect in normal cells. Moreover, it exerted inhibitory and lethal potential against five bacteria (MIC and MBC equal or below to 0.69 and 2.75 mg/mL for Gram-positive and Gram-negative bacteria, respectively). It is possible that the great bioactive potential of this decoction might be partially associated to its high levels in phenolic compounds (643.3 ± 18.9 µg/mg), particularly in rosmanol, a derivative of sageone, and hydroxycarnosic acid. In turn, although promising, the bioactivity of *S. farinacea* var. *Victoria Blue* was in general significantly lower than that of *S. apiana* and also contained modest levels of phenolic compounds (102.1 ± 0.7 µg/mg) comprised of moderate quantities of rosmarinic acid and glycosidic forms of the flavones apigenin and luteolin. The promising biological activities of *S. apiana and S. farinacea* var. *Victoria Blue* species herein found through in vitro methods suggest that decoctions of these species, and particularly that of *S. apiana*, might be useful for the application in the food and pharmaceutical industry, although their health-promoting potencies need to be further proved in vivo and pre-clinical studies.

## Figures and Tables

**Table 1:** Antioxidant activity (EC_50_, μg/mL) of *S. apiana* and *S. farinacea* var. *Victoria Blue* decoctions.

Assay	*S. apiana*	*S. farinacea* var. *Victoria Blue*	Standard		
			AA	BHA	Trolox
DPPH•	13.3 ± 1.1 ^a^	17.4 ± 5.5 ^a^	6.7 ± 0.7 ^b^		
Ferric reducing power	55.0 ± 5.6 ^a^	59.9 ± 3.6 ^a^		16.1 ± 2.0 ^b^	
TBARS	2.79 ± 0.1 ^a^	42.2 ± 0.6 ^b^			23.0 ± 1.0 ^c^
β-carotene bleaching inhibition	41.2 ± 1.6 ^a^	153.5 ± 2.2 ^b^			41.7 ± 0.3 ^a^

AA: Ascorbic acid; BHA: butylated hydroxyanisole; DPPH: 2,2-diphenyl-1-picrylhydrazyl; TBARS: thiobarbituric acid reactive substances. EC_50_: half maximal effective concentration, corresponding to 50% of extract antioxidant activity or 0.5 of absorbance in ferric reducing power assay. Means followed by the same letters in rows do not differ according to Tukey’s test (*p* < 0.05).

**Table 2:** Anti-inflammatory (EC_50_, μg/mL) and cytotoxicity properties (GI_50_, μg/mL) of *S. apiana* and *S. farinacea* var. *Victoria Blue* decoctions.

Effect	*S. apiana*	*S. farinacea* var. *Victoria Blue*	Standard
**Anti-inflammatory activity**			*Dexamethasone*
NO● production	49.9 ± 2.5 ^a^	80.8 ± 0.4 ^b^	16.0 ± 1.0 ^c^
**Cytotoxicity to tumor cell lines**			*Ellipticine*
HepG2 (hepatocellular carcinoma)	40.9 ± 3.3 ^a^	87.4 ± 5.4 ^b^	1.0 ± 0.2 ^c^
HeLa (cervical carcinoma)	57.3 ± 5.1 ^a^	77.8 ± 3.5 ^b^	2.0 ± 0.1 ^c^
MCF-7 (breast carcinoma)	60.2 ± 4.2 ^a^	59.8 ± 0.1 ^a^	1.0 ± 0.04 ^b^
NCI-H460 (non-small cell lung cancer)	245.7 ± 6.3 ^a^	279.5 ± 10.1 ^b^	1.0 ± 0.1 ^c^
**Cytotoxicity to non-tumor cells**			
PLP2 growth inhibition	361.7 ± 5.3 ^a^	335.4 ± 8.0 ^b^	3.0 ± 1.0 ^c^

Results of anti-inflammatory activity are expressed in EC_50_ values: sample concentration providing 50% of inhibition of nitric oxide (NO●) production. Cytotoxicity results are expressed in GI_50_ values (half maximal inhibition concentration) corresponding to the sample concentration achieving 50% of growth inhibition in human tumor cell lines or in liver primary culture PLP2. Means followed by the same letters in rows do not differ according to Tukey’s test (*p* < 0.05).

**Table 3:** Minimum Inhibitory Concentration (MIC) and Minimum Bactericidal Concentration (MBC) of *S. apiana* and *S. farinacea* var. *Victoria Blue* decoctions and nisin against selected test bacteria.

Bacteria	*S. apiana*		*S. farinacea* var. *Victoria Blue*		Nisin	
	MIC	MBC	MIC	MBC	MIC	MBC
**Gram-positive**						
*S. epidermidis*	0.34	0.69	8.50	8.50	<0.63	<0.63
*S. aureus*	0.69	0.69	1.06	2.12	<0.63	<0.63
**Gram-negative**						
*S. typhimurium*	2.75	2.75	>8.5	>8.5	0.5	0.5
*E. coli*	2.75	2.75	8.5	8.5	0.5	1.0
*P. aeruginosa*	2.75	2.75	>8.5	>8.5	1.0	1.0

MIC and MBC are expressed as mg/mL.

**Table 4:** Identification and quantification of the compounds identified in *S. apiana* and *S. farinacea* var. *Victoria Blue* decoctions determined by UHPLC-DAD-ESI-MS^n^.

NF	Rt	UVmax (nm)	[H–M]^−^	MS/MS Fragments (*m/z*)	Probable Compound	S. apiana *	*S. farinacea* Var. *Victoria Blue* *
1	1.5	275	149	103, 87, 131, 59	DimethylBA	-	5.9 ± 0.1
2	1.7	205	191	111, 173	Quinic Ac	5.0 ± 0.3	0.4 ± 0.01
3	3.6	280	197	179, 73, 153	Danshensu	D	D
4	4.4	261, 289	153	109	Protoc Ac	D	-
5	5.1	290sh, 324	353	191, 179, 135	*cis* 3-*O*-CQA	D	-
6	5.4	294sh, 322	353	191, 179, 135	*trans* 3-*O*-CQA	-	D
7	7.9	309	337	163	Coum Quinic Ac	D	0.3 ± 0.03
8	8.3	313	295	163	*p*-Coum Ac Pent	0.4 ± 0.04	-
9	8.8	290sh, 325	353	191	*trans* 5-*O*-CQA	-	0.6 ± 0.03
10	9.5	290sh, 325	353	173, 179, 191	4-*O*-CQA	5.5 ± 0.1	-
11	9.7	290sh, 323	179	135	Caffeic Ac	-	0.8 ± 0.0
12	9.8	314	325	265, 235, 163	Coum Hex	-	0.3 ± 0.0
13	11.8	311	337	191, 163	Coum Quinic Ac	D	0.2 ± 0.0
14	12.8	287sh, 324	367	173, 191	Fer Quinic Ac	-	D
15	13.0	309	225	207, 181, 165, 163	Coum Ac Der	1.8 ± 0.1	-
16	13.1	291sh, 311	637	351, 285, 193	Ferulic Ac Der	-	0.5 ± 0.0
17	13.5	274	571	527, 483, 439, 373	YA E (isom1)	-	8.4 ± 0.01
18	13.9	256, 267, 345	447	327, 357	Lut-*C*-Hex	-	3.2 ± 0.02
19	14.7	274	571	527, 509, 553, 483, 285	YA E (isom2)	-	4.5 ± 0.2
20	15.0	235, 277	539	297, 359, 377, 279, 315	YA D/isomer	-	3.9 ± 0.3
21	15.2	268, 336	431	311, 341, 269	Api-*C*-Hex	-	5.9 ± 0.6
22	15.6	285, 315	555	409, 391, 537, 511, 365	SA K	-	D
23	15.8	255, 350	463	301	Querc-*O*-Hex	14.6 ± 0.3	-
			593	285	Lut Rut	D	-
24	16.1	255, 266, 345	461	285	Lut-7-*O*-GlcA	-	15.8 ± 0.02
25	16.5	274	571	527, 409	YA E (isom3)	-	1.0 ± 0.08
26	17.6	268, 336	575	431, 341, 311, 513, 413	Api Hex HMG	-	6.3 ± 0.02
27	17.9	283	719	359, 539, 521, 341	Sag Ac (isom1)	D	2.1 ± 0.08
28	18.1	269, 329	431	269	Api-*O*-Hex	-	16.7 ± 0.05
29	18.3	238, 341	607	299, 284	Chrys-*O*-Rut	D	-
30	18.4	267, 337	445	269, 175	Api-*O*-GlcA	-	2.2 ± 0.01
31	18.6	270, 291, 326sh	717	555, 519, 475, 357	SA B (isom1)	-	D
32	18.7	284, 330sh	609	301	Hesperidin	41.3 ± 2.2	-
33	19.0	290sh, 328	359	161, 179, 197, 223	RA	56.8 ± 0.6	17.8 ± 0.1
34	19.5	285sh, 305	537	493, 295	CaffRA (isom1)	-	1.0 ± 0.04
35	19.8	278	719	521, 341, 359	Sag Ac (isom2)	-	2.2 ± 0.02
36	20.2	290sh, 333	537	493, 359, 375	CaffRA/ SA I (isom2)	D	-
37	20.6	267, 336	517	269, 473	Api malonyl Hex	-	2.5 ± 0.03
38	21.5	287sh, 320	373	179, 161, 135, 197, 355, 329	Methyl Rosmarinate	-	0.6 ± 0.02
39	21.8	290	491	163, 329, 119	Coumaric Ac Der	0.5 ± 0.01	-
40	22.4	281, 330sh	717	537, 357	SA B (isom2)	6.6 ± 0.4	-
41	23.7	199, 229, 287	361	299, 269, 281, 213, 343	Sageone Der	174.1 ± 14.1	-
42	24.3	275, 333sh	313	298, 283, 269	Cirsimaritin	25.9 ± 0.6	-
43	25.0	207, 237sh, 285	345	301, 271, 283	Rosmanol	192.4 ± 17.1	-
44	25.2	286	347	303, 273	Hydroxycarnosic Ac	69.7 ± 11.2	-
45	25.7	286	329	285	Carnosol	17.3 ± 0.7	-
46	26.3	262	331	287	Carnosic Ac	14.3 ± 0.7	-
47	26.4	277	301	271, 283	Tetrahydrohydroxyrosmariquinone	17.4 ± 0.2	-
					Total	643.3 ± 18.9	102.1 ± 0.7

* Values expressed as mg/g of extract; NF—Number of peak represented in Figure 1; D—Detected; Ac—Acid; Api—Apigenin; BA—Benzoic acid; Caff—Caffeoyl; CQA—Caffeoylquinic acid; Chrys—Chrysoeriol; Coum—Coumaroyl; Der—Derivative; Fer—Feruloyl; Glc—Glucoside; GlcA—Glucuronide; Hex—Hexoside; HMG—3-hydroxy-3-methylglutaroyl; Lut—Luteolin; Pent—Pentoside; Protoc—Protocatechuic; Querc—Quercetin; Rt—Retention time; Rut—Rutinoside; RA—Rosmarinic acid; Sag—Sagerinic; SA—Salvianolic acid; sh—shoulder; YA—Yunnaneic acid.
